# Immediate effects of Global Active Stretching on strength and flexibility: Randomised Controlled Trial

**DOI:** 10.17159/2078-516X/2024/v36i1a16618

**Published:** 2024-08-15

**Authors:** E Rodrigues, AR Pontes, G Brochado, I Bessa, P Carvalho, C Crasto

**Affiliations:** 1 Center for Rehabilitation Research (CIR), Escola Superior de Saúde, Politécnico do Porto, Rua Dr. António Bernardino de Almeida, nº 400 4200-072 Porto, Portugal; 2 Ministério da Educação – Agrupamento de Escolas de Alfena, Valongo, Portugal; 3 Escola Superior de Tecnologias de Saúde do Tâmega e Sousa, Paredes, Portugal; 4 Center for Translational Health and Medical Biotechnology Research (TBIO) – RISE – Health Research Network, Escola Superior de Saúde, Politécnico do Porto, Rua Dr. António Bernardino de Almeida, 400, 4200-072 Porto, Portugal; 5 Escola Superior de Saúde de Santa Maria, Porto, Portugal

**Keywords:** AKE test, FTF test, muscle chains, hamstrings

## Abstract

**Background:**

Global Active Stretching is a relatively recent yet little studied stretching method. It differs from the most popular methods by targeting muscle chains and integrating stretching with muscle contractions, which may eventually avoid the post-stretching reduction of strength that occurs in other methods.

**Objectives:**

To verify the immediate effects of Global Active Stretching on muscle strength and flexibility in individuals with short hamstrings.

**Methods:**

A single-blind randomised controlled trial was carried out on 30 volunteers with more than 20° in the active knee extension test, randomly assigned to three groups: the experimental group (Global Active Stretching exercise); the placebo group (Global Active Stretching initial position without stretching); and the control group (lying down). The active knee extension and fingertip-to-floor tests assessed hamstring and posterior chain flexibility. Hamstring and quadriceps strength were assessed using the peak torque evaluation in the Biodex System 4PRO^®^. Assessments took place before and immediately after the 15-minute intervention. The ANOVA and the paired t test were used (α = 0.05).

**Results:**

The experimental group had a significant increase in flexibility in both the fingertip-to-floor test (8.3 cm) and the active knee extension test (6.3°) when compared to the placebo and control groups (p < 0.05), while no differences in strength were observed (p > 0.05).

**Conclusion:**

Global Active Stretching immediately increased hamstring flexibility without significantly reducing muscle strength. Thus, individuals seeking to enhance their short-term flexibility can benefit from this programme in a single session without compromising performance.

Stretching is often used before sports practice and is one of the most effective ways to increase flexibility and relaxation however, its effects on subsequent performance are controversial. ^[1]^ Some studies have demonstrated that static stretching tends to cause an immediate decrease in strength, either isometric or isokinetic (mainly under concentric actions) ^[2]^, which in turn could challenge the concept of stretching in a warm-up because it would interfere with performance. ^[3–6]^ This issue is especially relevant when considering that in elite sports, small variations in performance can make a difference. ^[7]^

In recent years, a method of self-stretching exercises, also called Global Active Stretching (SGA^®^), was created for athletes and is currently used in the general population. It has emerged as an alternative to segmental stretching despite its effects being little studied. SGA^®^ involves the performance of progressive self-stretching exercises based on the global stretching of muscle chains, in which breathing plays a crucial role. ^[8]^ The stretching is carried out progressively, starting with smooth movements with a slow angular displacement speed (potentially reducing stress on muscle spindles and minimising the reflex muscle stretch response, which could affect neural activation), in which its progression and maintenance time in each new joint range are determined by whether compensations appear. The choice of exercises is based on each individual’s needs and according to their daily or sporting activities. As the name suggests, this is a method in which muscle activation, both of the antagonists of the stretched muscles and of the stretched muscles themselves, is required, promoting joint stability and neuromuscular control, and can also contribute to preserving strength while working on flexibility. ^[8]^

In clinical practice, SGA^®^ is effective in postural correction ^[9]^ and flexibility enhancement. ^[10]^ Regarding muscle strength and jump performance, the few studies investigating this relationship focused only on the long-term effect and found an increase in muscle strength and vertical jump. ^[11, 12]^ Thus, the aim of this study was to verify the immediate effects of the SGA^®^, more specifically the “frog in the air” exercise, on strength and flexibility in asymptomatic participants with hamstring shortening.

## Methods

### Study design

A single-blind (participants blinded) randomised controlled trial was performed to determine the effect on flexibility and strength of the “frog in the air” exercise used by the SGA^®^ method in three groups: experimental, control, and placebo. These variables were measured in two moments: before (M0) and immediately after intervention (M1).

### Participants

Thirty asymptomatic participants of both sexes, between the ages of 19 and 31 years, selected in a non-probabilistic and voluntary manner, were assigned, in order of arrival, to three groups: the experimental group (EG), which was submitted to the SGA^®^; the placebo group (PG), in which the participants were placed in the same exercise starting position; and the control group (CG), which stayed lying down. The sample included individuals with knee flexion angles greater than 20° in the active extension knee test. Those participants who had performed segmental or global stretching on a regular basis, those with a history of orthopaedic and/or neurological pathology, symptomatic participants, and female participants who were in the ovulatory phase were excluded ^[2]^. Participants were asked not to engage in any strenuous activity or change their normal movement pattern in the 48 hours preceding the testing session.

G*Power software (version 3.1.9.4; Heinrich Heine Universität Düsseldorf, DE) was used to calculate the required sample size to detect differences between the experimental and placebo groups concerning the changes between M0 and M1 (the difference variable). An effect size of 1.46 for the active knee extension test and 0.29 for the knee flexor peak torque was found using the data from the first ten participants in each group. According to these results, the necessary sample size to achieve a power of 0.80 with an α = 0.05 was nine participants per group to identify differences in flexibility and 183 to detect differences in knee flexor strength. Since the main goal was flexibility outcomes and the effect size on strength was small, only 10 participants in each group were assessed.

### Ethical approval

This study was conducted at the Rehabilitation Research Centre of the School of Health of the Polytechnic of Porto. It was approved by the ESS-P ethics committee (process no. 0787, dated July 30, 2018). All participants signed the informed consent form according to the standards and principles of the Declaration of Helsinki (2013).

### Procedures

#### Familiarisation session

Before data collection (seven days before), a pilot study was conducted on five participants with similar characteristics to those of the sample, but not included in this, to allow the research team to become familiar with the procedures.

After collecting anthropometric data, flexibility was assessed, starting with the active knee extension (AKE) test ^[13]^ and the fingertip-to-floor (FTF) test. ^[14]^ Three repetitions of each test were performed, and the mean was calculated. The participants rested for one minute between attempts and three minutes between flexibility tests. The strength of the quadriceps and hamstrings was then assessed on an isokinetic dynamometer.

Immediately after the assessments, interventions were implemented according to the participants’ group. Then, the assessment tests took place in the same order as they were initially conducted. There were no breaks between intervention and re-evaluation procedures, apart from the time needed for preparation.

All measurements were performed in the same session, without warm-up, and all participants were assessed under the same conditions (in the morning, at the same place, at the same temperature) and by the same researcher.

#### Anthropometric data

Participants’ mass and height were collected using a Seca 760 scale (Medical Scales and Measuring Systems^®^, Birmingham, United Kingdom), accurate to 1kg, and a Seca 222 stadiometer (Medical Scales and Measuring Systems^®^, Birmingham, United Kingdom), accurate to 1mm, respectively.

#### Flexibility assessment

Flexibility assessment was performed through two tests: the AKE test ^[13]^ and the FTF. ^[14]^

The AKE test is considered the gold standard for measuring hamstring muscle length. ^[13]^ In this study, the knee extension angle during maximum muscle lengthening was measured using a universal goniometer (Baseline Hires, 12”). The axis of the goniometer was placed over the lateral femoral condyle, the stationary arm lined up with greater trochanter, and the moving arm lined up with lateral malleolus direction. Participants were asked to lie supine, and the tested limb was positioned at 90° of hip flexion with the contralateral limb in full extension. In this position, the participants actively extended the knee until they felt moderate resistance.

The FTF test analyses posterior chain flexibility, providing information about the spine and pelvis mobility when the participant leans forward in the orthostatic position. ^[14]^ It is a simple and quick test to perform that has been described as having very high inter-measurement reliability (ICC = 0.99). ^[14]^ Participants were asked to stand barefoot in an orthostatic position with their feet together, or, in the case of excessive valgus, with their knees together. After assuming this position, they were asked to lean the trunk forward as far as they could, with their neck relaxed, while keeping their knees, arms, and fingers fully extended. This position was maintained long enough to allow measurement of the distance (cm) between the third finger of the participant’s right hand and the ground using a tape measure. The participants who easily touched the ground were positioned on a platform approximately 30 cm high, and the measurement was repeated, but counting negative values from the top of the platform. As in the previous test, three repetitions were performed, and the mean was calculated.

#### Assessment of muscle function

The Biodex System 4 Pro^®^ (Biodex Corporation, Shirley, New York, USA) dynamometer assessed hamstring and quadriceps peak torque (PT). The Biodex is an instrument that presents high validity and reliability ^[15]^ and has been regarded as the gold standard for assessing muscle function in a laboratory environment. The participants sat on the isokinetic dynamometer with the hip joint positioned at 80° of flexion, the axis of the device lined up with the axis of their knee joint. The resistance was placed on the distal third of their leg, two centimetres above the lateral malleolus. The participants were held in this position using bands to limit the trunk, pelvis, and thigh movements. Before starting the test, the procedures were explained, and a brief warm-up of ten consecutive submaximal concentric contractions was performed to familiarise the participants with the desired procedure. The test amplitude was 90° (90° – 0° and 0° – 90°), and six repetitions were performed at a speed of 60°/s. Data were collected on the dominant limb only, which was identified by asking participants which leg they used to kick a ball. Participants who felt pain in the hamstring or quadriceps muscles during the testing procedure would have been excluded from the study.

#### Intervention

**Fig 1:**
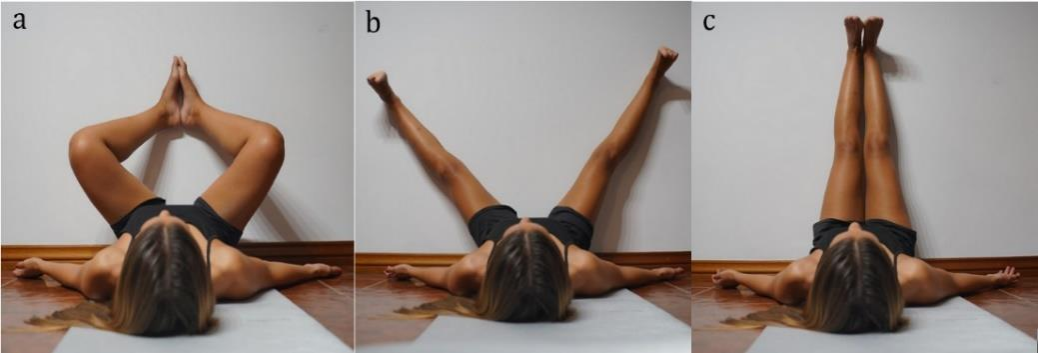
“Frog in the air” exercise (a) Initial phase: hip flexion and abduction and knee flexion; (b) Intermediate phase: knee extension; (c) Final phase: hip adduction

Participants performed the “frog in the air” exercise in the EG. This exercise has a 15-minute duration and specifically targets the large posterior muscle chain. ^[8]^ The participants were placed supine with the lumbar spine completely supported on the ground and aligned with the occiput. The upper limbs were placed at 45° abduction with elbows in extension and palms facing upward. The lower limbs started in hip flexion, maximum abduction and lateral rotation and knee flexion with the soles of the feet together against the wall (Figure 1a). A specific breathing pattern is a fundamental part of the approach and was instituted during the exercise. Participants were instructed to inhale air through their nose, enlarging the lower rib region, as part of the inspiration process. During exhalation, the upper chest descended while the abdomen protruded. Subsequently, the participants were invited to perform knee extension slowly and gradually, while ensuring that the lower limbs remain abducted and the initial positions of the head, trunk, and upper limbs are maintained. (Figure 1b). If any form of compensation occurs, the advancement must cease. If the participant experiences any discomfort, it is imperative that they cease the activity and engage in mild hamstring isometric contractions for three seconds following a deep breath. Once knee extension was achieved, they were asked to gradually bring their knees closer together, keeping them slightly rotated outward. Ankle dorsiflexion and toe extension were successively increased (Figure 1c). ^[8]^ The phases have no specific duration. The exercise is done progressively, according to the participant’s ability, aiming to reach the final position (c) in 15 minutes.

To optimise muscle stretching and prevent compensations during the exercise, the researcher used verbal cues to request the maintenance of alignment and the necessary postural corrections. Participants were instructed to perform gentle inhales followed by prolonged exhales, lowering the ribs as much as possible and protruding the abdomen to stretch the inspiratory muscle chain. ^[8]^ Before the study, a training session was held to inform the participants about the exercise components and the procedures for their implementation. The researcher who guided the sessions was experienced in this area.

Participants in PG stayed in the initial position of the frog in the air exercise for 15 minutes. This position does not strain the posterior chain muscles enough to qualify as a stretching intervention (Figure 1a). CG participants remained in a lying supine position for 15 minutes with both legs extended. This position was selected as it keeps the torso in the same position as the EG and does not strain the hamstring muscle.

### Statistical analysis

Statistical analysis was performed using the IBM^®^ Statistical Package for the Social Sciences^®^ 28.0 (SPSS) software with a 0.05 significance level. The one-way ANOVA followed by Tukey’s test was used for a between-groups comparison and the paired t-test for a within-groups comparison (M0 – M1). The normality assumption was checked through the Shapiro-Wilk test. The mean and standard deviation were used as descriptive statistics. The effect size was determined using Cohen’s *d* formula, with the common magnitude being small (= 0.20 – 0.50), medium (> 0.50 – 0.80), and large (> 0.80) effects. ^[16]^

## Results

**Fig 2:**
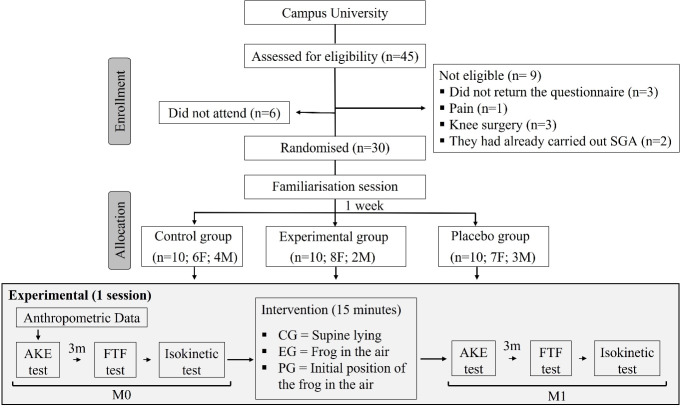
Sample diagram and flow of participants through experimental sessions of the study. F, female; M, male; AKE, active knee extension; FTF, finger to floor; CG, control group; EG experimental group; PG, placebo group; M0, before intervention; M1, immediately after intervention.

Out of the 45 participants who volunteered, nine were excluded for not meeting the sample selection criteria, and six did not attend on the scheduled data collection date. As a result, 30 participants (both sexes) were randomly assigned to three groups of ten individuals each (Figure 2).

Table 1 describes the participants’ demographic (age) and anthropometric (weight, height, and body mass index) data. The groups were similar with respect to these variables. (p > 0.05).

### Flexibility evaluation

**Fig 3:**
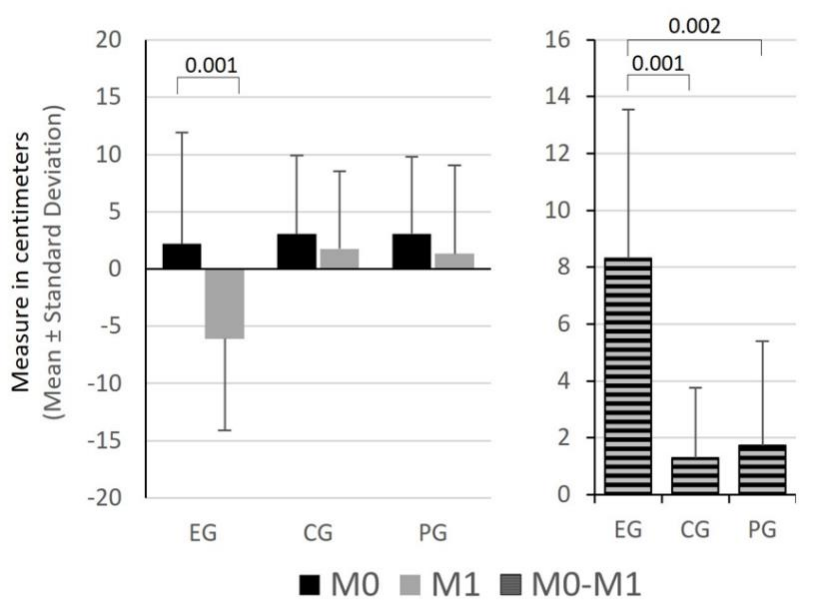
Comparison of mean values and standard deviation of flexibility in the Fingertip-to-floor test and between the variable difference in the different groups. EG, experimental group; CG, control group; PG, placebo group; M0, before intervention; M1, immediately after intervention

**Fig 4:**
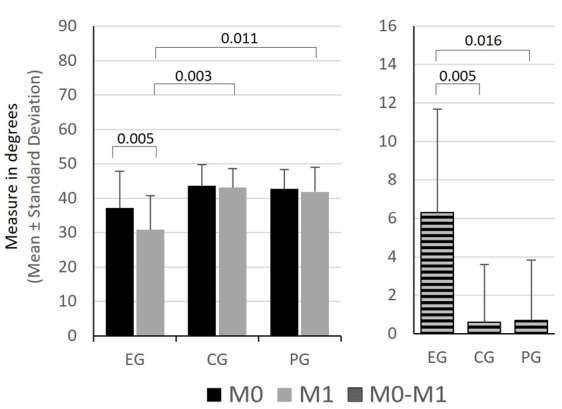
Comparison of mean values and standard deviation in the active knee extension test and between the variable difference of flexibility in the different groups. EG, experimental group; CG, control group; PG, placebo group; M0, before intervention; M1, immediately after intervention

Only the EG showed changes in the flexibility outcomes. The FTF distance (Figure 3) and the knee joint flexion on the AKE test (Figure 4) both decreased significantly (p = 0.001 and p = 0.005, respectively).

Differences were also observed in the AKE test in M1, where EG showed significantly lower values (higher flexibility) than the CG (p = 0.003) and the PG (p = 0.011) (Figure 4).

In M1, no differences between groups were observed regarding the FTF distance; however, the EG showed significantly higher changes between moments than the other groups (EG vs CG: p = 0.001 and EG vs PG: p = 0.002) (Figure 3).

In the AKE test, the EG also showed significantly higher changes between moments than the other groups (EG vs CG: p = 0.005 and EG vs GP: p = 0.016) (Figure 4).

### Strength evaluation

Regarding strength, as shown in Table 2, no significant differences were found between groups or between moments in the quadriceps or the hamstring peak torque.

## Discussion

A single 15-minute SGA^®^ session of the “frog in the air” exercise has proven to be effective in increasing hamstring and posterior chain flexibility since significant differences were detected between the groups in the two tests analysed, with a mean of approximately 6.3° in the AKE test and 8.3 cm in the FTF test. To our knowledge, the extent to which the knee range of motion should increase in the AKE test and how much should decrease the distance from the third finger to the ground in the FTF test, after stretching, to be considered clinically relevant has not yet been determined and must depend on the desired functional outcome and the person’s age and lifestyle. However, in the study by Oh and Kang, ^[17]^ which compared Proprioceptive Neuromuscular Facilitation with static stretching, the results of the latter (AKE test = 6.7° and FTF = 7.2 cm) were similar to those obtained in this study, which suggests that SGA^®^ is equally effective in improving flexibility.

These results were expected because the FTF test measures posterior chain flexibility and the “frog in the air” exercise targets posterior muscles of the spine, pelvis, and lower limbs. ^[8, 10]^ The technique effect was also shown in the AKE test since the hamstrings are a part of the posterior chain. ^[8]^ Mechanical factors related to the continuity of the connective tissue chain may explain these changes, but above all, there may be neural factors associated with an increase in global tolerance to stretching. ^[18]^ These findings can also be supported by the fact that this method promotes the reciprocal inhibition phenomenon, which stimulates the proprioceptive (via activation of neuromuscular spindles, Golgi neurotendinous organs, and joint receptors), exteroceptive (via activation of skin mechanoreceptors), and auditory (via verbal command that activates the reflex connections of the auditory pathway with the motor nuclei of the brainstem) pathways. ^[3]^

In this study, Global Active Stretching had no effect on force production capacity at 60^o^/s velocity, as no significant differences in muscular force were found between groups or between moments in any of the studied variables. Using static stretching as a model, it was reasonable to anticipate an immediate reduction in force as an acute effect. ^[4–6]^ The lack of differences may suggest that this type of stretching, while increasing flexibility, does not affect strength. These findings could be related to the unique characteristics of this stretching technique, which adopts a comprehensive approach by focusing on muscular chains rather than isolated muscles, thus involving several joints and muscle groups simultaneously. ^[8]^ Therefore, in principle, the stretching force is evenly spread throughout the several muscles in the chains, decreasing its intensity in each muscle ^[19]^ and consequently minimising the impact on peak torque. Nevertheless, it was crucial to include a static stretching group to substantiate this hypothesis. The low intensity and longer duration used in this approach may have a more pronounced effect on the connective tissue/fascia due to its higher density. Consequently, the effect on the myotendinous unit will be less, reducing the impact on muscle strength. ^[8]^ On the other hand, these changes may facilitate a gradual neural adaptation, allowing the central nervous system to adjust to new ranges of movement without significantly inhibiting muscle activation. Furthermore, the fact that SGA^®^ combines stretching with muscle contractions can also contribute to a lesser influence on strength.

Some limitations may have contributed to these results. The methodology used may also be associated with the lack of changes in strength. The sample size may have played a role in the absence of significant differences observed between the groups in relation to muscle strength. As previously described in the methodology, to achieve a test power of 0.80 with the small effect size observed (Cohen *d* = 0.29) for this objective, it would be necessary to assess 183 people in each group. The low effect size also means that even if there were significant differences, their magnitude could point to low clinical relevance regarding muscle strength. Another possibility is the time interval between the SGA^®^ and the strength assessment, which occurred approximately seven minutes post-intervention and may have contributed to a loss of effect size. Silva and Souza et al. ^[20]^ found that this effect was no longer present five minutes after stretching. However, Nakamura et al. ^[21]^ observed that the reduction in strength was maintained for up to 10 minutes after five minutes of static stretching.

These results are important because they suggest this technique is effective at immediately increasing hamstring and posterior chain flexibility without apparently compromising muscle strength. In this way, SGA^®^ could be an alternative strategy for specific populations who want to increase flexibility in the short term without compromising performance. However, the effects and feasibility of their application to these special populations would have to be assessed, in future studies.

Another advantage of this strategy is its direct and indirect effects on regulating the movement required to perform the exercises, thus improving motor control.

In a future study, it would be relevant to incorporate a static stretching group and include a population of athletes. It would also be important to use a larger sample and explore the duration of the SGA^®^ effect.

## Conclusion

According to our results, a single 15-minute session of SGA^®^ appears to increase flexibility in both the fingertip-to-floor test and the active knee extension test without apparently affecting hamstring or quadriceps strength (measured at a speed of 60°/s).

## Figures and Tables

**Table 1:** Mean values and standard deviation of the variables age, weight, height, and body mass index (BMI) in the different groups

	EG (n = 10)	CG (n = 10)	PG (n = 10)	P value
Age (years)	25.7 ± 3.7	23.4 ± 3.0	23.3 ± 3.0	0.20
Weight (kg)	72.3 ± 12.3	68.6 ± 9.3	67.6 ± 8.9	0.57
Height (cm)	170.9 ± 8.5	169.6 ± 11.3	167.4 ± 10.1	0.74
BMI (kg/m2)	24.7 ± 3.8	23.9 ± 2.3	24.1 ± 2.2	0.80

Data are expressed as mean ± SD. EG, experimental group; CG. control group; PG. placebo group.

**Table 2:** Mean, standard deviation, test value, and p-value of the Quadriceps peak torque (QPT) and Hamstring peak torque (HPT) variables at each moment in the different groups

		EG	CG	PG	P value
**QPT (N·m)**	M0	145.7 ± −0.8	138.4 ± 46.9	128.4 ± 39.2	0.85
	M1	148.1 ± 41.0	140.3 ± 44.2	128.8 ± 37.7	0.76
	Diff*	−2.4 ± 9.2	−1.8 ± 22.2	−0.5 ± 15.5	0.85

**HPT (N·m)**	M0	71.7 ± 17.6	65.4 ± 23.4	59.7 ± 20.9	0.63
	M1	76.0 ± 22.2	65.7 ± 22.9	61.6 ± 21.1	0.42
	Diff*	−4.3 ± 7.8	−0.3 ± 9.7	−1.9 ± 8.5	0.47

Data are expressed as mean ± SD. N·m, newton-meter; EG, experimental group; CG. control group; PG. placebo group; M0, before intervention; M1, immediately after intervention; Diff = M0–M1.

* Changes within groups were not statistically significant (p > 0.05).
